# What Structural Racism Is (or Is Not) and How to Measure It: Clarity for Public Health and Medical Researchers

**DOI:** 10.1093/aje/kwac112

**Published:** 2022-07-05

**Authors:** Lorraine T Dean, Roland J Thorpe

**Keywords:** discrimination, measurement, racial inequities, structural racism

## Abstract

Interest in studying structural racism’s impacts on health has grown exponentially in recent years. Across these studies, there is much heterogeneity in the definition and measurement of structural racism, leading to mixed interpretations of structural racism’s impact on health. A precise definition of structural racism can offer conceptual clarity to inform what mechanisms to investigate and is imperative for conducting high-quality research on it and dismantling it. In this commentary, we trace the evolution of the definitions of structural racism and suggest ways in which the measurement of structural racism should move forward given these definitions.

## Abbreviations

FOA: Funding Opportunity Announcement
PUMA: Public Use Microdata Area

**Figure 1:**
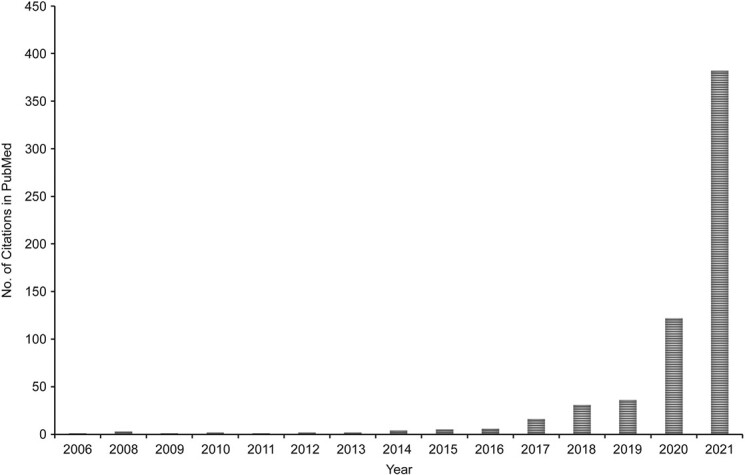
Number of publications on structural racism and health in PubMed as of December 31, 2021.

The horrific events of police brutality against African-American men and women, particularly African-American men, heighten awareness of the role of racism in the United States. One of the key outcomes of this racial and social unrest is the pointed discussion about how racism permeates all structures in society. Structural racism has now been named a public health issue and remains a fundamental cause of health inequities (1). Schools of medicine and public health now explicitly acknowledge racism as a public health problem (2, 3) and the need to dismantle it as being within the domain of the responsibility of health and medicine disciplines. There has been a burst of health studies focusing on structural racism and health: A PubMed query (National Library of Medicine, Bethesda, Maryland) of papers on (“structural racism” AND “health”), as of the end of 2021, showed over a 50-fold increase in citations in just the past 5 years (Figure 1).

For the first time ever, funding opportunities have arisen to explicitly advance the science of structural racism and health (4). For example, in 2021, the National Institutes of Health issued requests for proposals focused on structural racism’s impacts on health (“Understanding and Addressing the Impact of Structural Racism and Discrimination on Minority Health and Health Disparities” (Funding Opportunity Announcement (FOA) no. RFA-MD-21-004) and “Measures and Methods to Advance Research on Minority Health and Health Disparities-Related Constructs” (FOA no. PAR-22-072)). Around that same time, the Robert Wood Johnson Foundation (Princeton, New Jersey) issued several calls for community-engaged research on structural racism and health, all of which represent the shift of influence to racism as a public health problem that funders will support.

However, across these publications and funding opportunities, there is much heterogeneity in the definition and measurement of structural racism in health studies. Few offer explicit definitions, which leads to conceptual inconsistencies and, consequently, varying measurements. Some academics have observed that “health equity tourists” have infiltrated work on structural racism, contributing to greater confusion by untrained scholars who mischaracterize health equity concepts (5, 6). Mismeasurement of the construct has meant that the effects of structural racism are absorbed in measurements of interpersonal discrimination, leading to inflated estimates of interpersonal racism and interventions that overly focus on individual-level solutions (7).

In epidemiology, accurate measurement requires clear and precise definitions of what we are trying to measure (8). Precise definitions offer conceptual clarity that informs what mechanisms we wish to investigate (9); thus, having a clear and precise definition of structural racism is imperative to conducting high-quality research on it and dismantling it.

In this commentary, we trace the evolution of the definitions of structural racism, offering conceptual clarity toward better measurement.

## WHAT STRUCTURAL RACISM IS NOT

In the case of structural racism, it’s important to disentangle its influences from racism at other levels (7, 10). Dr. Camara Jones’ seminal 2000 paper using the “gardener’s tale allegory” (10) delineates 3 distinct levels of racism: 1) internalized racism, 2) interpersonal (or “personally mediated,” which could also be inclusive of aversive (11, 12)) racism, and 3) institutional racism. Dr. Jones’ work provided a provocative discourse on how the field of public health should think about racism and discrimination and their connections to health, but the term “structural racism” was absent from the paper. The concept of structural racism was not wholly absent from health research literature at the time, with a few scholars identifying a need to explore it distinctly, often wrapped in the larger construct of structural discrimination, which included additional axes of power outside of race/ethnicity (8). Instead, Jones’ paper referred to institutional racism as the highest level of racism, representing an unequal distribution of resources and power that manifests as normative or structural barriers to advancement.

Given the historical dearth of the term “structural racism” in public health literature, the concepts of structural racism and institutional racism are often conflated, but scholars more recently have clarified and affirmed that they are not the same thing (7, 13–15). This confusion may arise because of the evolution of the term, as well as the similar-sounding concept of “systemic racism,” which is used to qualify the existence and extent of racism in systems of power but is not generally referenced as a measurable construct.

## WHAT STRUCTURAL RACISM IS

The initial paper in PubMed that featured structural racism (16) focused on the health of migrant workers in the United States and, while mentioning structural racism in the abstract, did not explicitly define it. Five years later, in a paper reviewing the links between structural racism and health inequities, Drs. Gil Gee and Chandra Ford wrote,

Structural racism is defined as the macrolevel systems, social forces, institutions, ideologies, and processes that interact with one another to generate and reinforce inequities among racial and ethnic groups (Powell 2008). The term *structural racism* emphasizes the most influential socioecological levels at which racism may affect racial and ethnic health inequities. Structural mechanisms do not require the actions or intent of individuals (Bonilla-Silva 1997). (1, p. 3)

Drs. Gee and Ford did not initially differentiate between institutional and structural racism; however, that same text hints that there is something about the interactive nature of structural racism that goes beyond other siloed forms of racism that exist within an individual, an interpersonal relationship, or an institutional system:

Research on structural racism should not only focus on independent effects but also should address interactions among multiple forms of racism. Further, it is likely that forms of racism may reinforce one another, and efforts to dismantle one system may yield little effect without simultaneous efforts on another system... The study of single forms of racism would lead to an incomplete understanding and, potentially worse, biased estimates. (1, p. 13)

Later, Dr. David Williams et al., in a 2019 paper, confirmed that the terms are used interchangeably in the social science literature, but also noted that structural racism is reinforced and supported by multiple societal systems, including the housing, labor, and credit markets and the education, criminal justice, economic, and health-care systems.

We use the terms institutional and structural racism, interchangeably, consistent with much of the social science literature (13, 55, 106). Institutional racism refers to the processes of racism that are embedded in laws (local, state, and federal), policies, and practices of society and its institutions that provide advantages to racial groups deemed as superior, while differentially oppressing, disadvantaging, or otherwise neglecting racial groups viewed as inferior (13, 104). (7, p. 107)

Even here Dr. Williams hints that there are multiple institutions of racism, which in totality are embedded within a larger structural racism system where these institutions interact. Williams also differentiates “cultural racism,” which reflects ideologies around the inferiority of a racial/ethnic group and that sets societal norms that lead to acts of discrimination (7).

The most contemporary and updated definition of structural racism was offered by Drs. Zinzi Bailey and Mary Bassett, in 2 callout boxes that gave a specific definition and distinction:

Many academics use structural racism and institutional racism interchangeably, but we consider these terms as two separate concepts.

Structural racism refers to “the totality of ways in which societies foster [racial] discrimination, via mutually reinforcing [inequitable] systems… (e.g., in housing, education, employment, earnings, benefits, credit, media, health care, criminal justice, etc.) that in turn reinforce discriminatory beliefs, values, and distribution of resources”, reflected in history, culture, and interconnected institutions^9^. This definition is similar to the “über discrimination” described by Reskin^10^.

Within this comprehensive definition, institutional racism refers specifically to racially adverse “discriminatory policies and practices carried out… [within and between individual] state or non-state institutions” on the basis of racialised group membership^9^. (14, p. 1455)

Structural racism involves interconnected institutions, whose linkages are historically rooted and culturally reinforced. (14, p. 1454)

Later, they admit that there is no officially agreed-upon definition; however, they emphasize that even with no single definition, structural racism is still distinct from institutional racism.

There is no “official” definition of structural racism—or of the closely related concepts of systemic and institutional racism—although multiple definitions have been offered^3–7^. (13, p. 768)

Appreciating the evolution of these terms, we adopt the Bailey and Basset definition (14) as the most contemporary definition that should be followed by scientists investigating structural racism and health: *Structural racism* represents the totality of ways in which multiple systems and institutions interact to assert racist policies, practices, and beliefs about people in a racialized group. That definition retains its distinction from: *institutional racism*, which is racism within a particular type of institution; *systemic racism*, which is a descriptive term about racialized systems of power; *racial discrimination*, which is action that stems from racist beliefs; and *cultural racism*, which reflects the ideologies and societal norms about a particular racial/ethnic group. Structural racism encompasses all these aspects and adds that these institutions, norms, and actions *interact* to influence health.

## STRUCTURAL RACISM MEASURES

Measurements of structural racism have been as equally heterogenous as its definitions (17). The most common measures have been in a single domain of racism, namely residential segregation (17), which, as applied to the Bailey and Basset contemporary definition of structural racism, we argue better represents a form of institutionalized racism than structural racism. Scholars have similarly mislabeled other measures of racism within an institution—for example, political representation or incarceration—as structural racism; however, we believe that to transcend as a measurement of structural racism, a key component is to capture the *interactive* effects across multiple institutions. There have been several advancements in this area.

In one of the earliest empirical papers on structural racism, Dr. Maeve Wallace et al. assessed 5 domains of structural racism (educational attainment, median household income, employment, imprisonment, and juvenile custody) at the state level (18). Across all US states, more unemployment inequity was associated with a 5% increase in Black infant mortality, and less racial inequity in education was associated with an approximately 10% reduction in the Black infant mortality rate, while none of the structural racism measures were associated with White infant mortality (19). A published measure developed by Dr. Alicia Lukachko et al. (20) included assessment of 4 separate domains (political participation, employment and job status, educational attainment, and judicial treatment) at the state level. Their cross-sectional study showed that Black people living in states with high levels of structural racism were more likely to report past-year myocardial infarction than those living in states with low structural racism, while these associations were null or inverse among White people in these states (20). While these papers novelly captured the role of multiple institutions, these studies assessed single indicators in separate regression models, which may not fully capture the interacting or overlapping influences of societal structures. As recommended by scholars Dr. Paris Adkins-Jackson (15) and Dr. Jaquelyn L. Jahn (21), structural racism’s multidimensional and interactive qualities are best captured by index measures.

Several novel indicators of structural racism that were explicitly intended to capture those interactive features highlight the use of index measures to capture the interactive effects of structural racism across multiple institutions. Recently, in a paper by Dr. Tongtan Chantaratat, senior-authored by Dr. Rachel R. Hardeman (22), these scholars introduced a multidimensional measure of structural racism in Public Use Microdata Areas (PUMAs). PUMAs include various types of geographic designations that can range from single census tracts to groups of counties with a state. Their measure included indicators for Black-White residential segregation and inequities in education, employment, income, and home ownership, which they applied to coronavirus disease 2019 (COVID-19) vaccination. While a measure based on PUMAs is limited in use for guiding county or regional laws, practices, or programs for dismantling structural racism, it may be valuable for state or federal decision-makers, as well as for tracking populations over time.

**Table 1:** Challenges and Suggested Recommendations for Structural Racism Measures

**Domain**	**Challenge**	**Recommendations**
Definition	Conflation of structural racism with other levels and types of racism	Identify the level(s) at which racism is operating and be precise about which are (or are not) being measured.
		Agree on a definition and be accountable to using that definition.
Measurement	Failure to capture multidimensional and interacting elements of racism	Use index measures (15, 21).
	Existing measures’ having applicability to a limited number of racial/ethnic groups	Develop measures with indicators that are specially targeted to how structural racism presents itself in other racial/ethnic groups.
	Need for better assessment across units of time and space	Use psychometric evaluation to test measures for relevance over historical eras and life-course time (15, 24, 25).
		Develop structural racism measures for use at different levels of geography of exposure or unit of intervention.
Interpretation	Choosing which structural racism measure to use	Use theory and frameworks to guide the choice of measure for your study.
	Studies erroneously labeled as measuring structural racism leading to mixed interpretation of structural racism’s impact on health	Prior to conducting a study or review, establish and state definitional criteria for what qualifies as structural racism and what does not.

Another structural racism index measure, developed by Dr. Geoff Dougherty et al., used a 5-domain index to capture the compounding effects of county-level structural racism in education, housing, employment, criminal justice, and health care and applied it to body mass index (weight (kg)/height (m)^2^) (23). That analysis found that county-level structural racism was associated with lower body mass index in White adults but higher body mass index in Black adults. This index measure captures the interactive nature of racist structures and institutions and was developed for use by county leadership. However, like the previous measures, it is a cross-sectional measure that may not reflect the dynamic and changing nature of racist structures over time. We promote the recent work suggesting that these cross-sectional measures might be improved upon by considering how structural racism measurements can embrace a life-course approach, since the ways in which racism operates in society is dynamic over the course of a person’s life (15, 24, 25).

Each of these measures is calculated at a different geographic level. Geographic level is important when considering structural racism as an *exposure* (as well as whether we even have data with which to measure it at a particular geographic level) or the geographic levels at which structurally racist policies (e.g., laws, practices, or programs) are enacted and when considering how to intervene upon structural racism. In some cases, state policies may represent important exposures that are readily measurable, but it may take interventions at the federal or county level to dismantle them. That said, there is no one gold standard geographic unit of measurement for structural racism, which is why we highlight the work of scholars who have assessed structural racism at county, state, and other geographic levels.

## FUTURE DIRECTIONS

We view each of these as promising examples of the future of structural racism measurement that set a strong foundation for improved measurement of this distinct construct. Our recommendations for improving structural racism measures appear in Table 1. While each of the measures has limitations, each is also useful and valuable, and the choice of use of these and future measures should be guided by theory and the framework of studies for which their use is proposed. We also note that existing measures are largely based on Black and White US populations, but there are other racial and ethnic groups for which measures should be tailored and developed. We now need to move the field forward to ensure that structural racism measures are relevant and useful over periods of time, across racial and ethnic groups, and across the life course and are flexible enough to accommodate the geographic levels at which policies to dismantle structural racism are made. Index measures, or others that capture the interactive effects of racism at various levels, should be used. Having a consistent, agreed-upon definition is the first step toward improving future measures. Researchers and reviewers of structural racism measures should discern whether studies meet the definitional criteria of structural racism (i.e., evaluate whether the study captures the interaction and multidimensional properties of racism across intersecting institutions), so that we can adequately distinguish its impacts on health.

## CONCLUSION

Lack of conceptual and methodological distinction of structural racism from other forms of racism has led to inconsistent measurement of this construct. Our paper traces the evolution of the definitions of structural racism and advocates the use of the most contemporary definition of structural racism—that it is the totality of forms of racism operating across intersecting institutions—which honors the history of the literature on structural racism and brings a nuanced understanding of this work. Finally, we recommend that new or revised measures of structural racism start with clear definitions of the construct, use index measures on a set of indicators defined by a theoretical approach, adapt to other racial/ethnic groups and geographic levels, and take a life-course approach to measurement. Adopting these practices may better enable us to assess the impact of structural racism on health more precisely and how, when, and where we need to intervene to dismantle it.
